# Neurobiology of Recovery of Motor Function after Stroke: The Central Nervous System Biomarker Effects of Constraint-Induced Movement Therapy

**DOI:** 10.1155/2020/9484298

**Published:** 2020-06-15

**Authors:** Auwal Abdullahi, Steven Truijen, Wim Saeys

**Affiliations:** ^1^Neurological Rehabilitation Unit, Department of Physiotherapy, Bayero University Kano, Nigeria; ^2^Department of Rehabilitation Sciences and Physiotherapy, University of Antwerp, Movant, Wilrijk, Belgium

## Abstract

Recovery of motor function after stroke involves many biomarkers. This review attempts to identify the biomarker effects responsible for recovery of motor function following the use of Constraint-Induced Movement Therapy (CIMT) and discuss their implications for research and practice. From the studies reviewed, the biomarker effects identified include improved perfusion of motor areas and brain glucose metabolism; increased expression of proteins, namely, Brain-Derived Neurotrophic Factor (BDNF), Vascular Endothelial Growth Factor (VEGF), and Growth-Associated Protein 43 (GAP-43); and decreased level of Gamma-Aminobutyric Acid (GABA). Others include increased cortical activation, increased motor map size, and decreased interhemispheric inhibition of the ipsilesional hemisphere by the contralesional hemisphere. Interestingly, the biomarker effects correlated well with improved motor function. However, some of the biomarker effects have not yet been investigated in humans, and they require that CIMT starts early on poststroke. In addition, one study seems to suggest the combined use of CIMT with other rehabilitation techniques such as Transcortical Direct Stimulation (tDCs) in patients with chronic stroke to achieve the biomarker effects. Unfortunately, there are few studies in humans that implemented CIMT during early poststroke. Thus, it is important that more studies in humans are carried out to determine the biomarker effects of CIMT especially early on poststroke, when there is a greater opportunity for recovery. Furthermore, it should be noted that these effects are mainly in ischaemic stroke.

## 1. Introduction

Stroke is a leading cause of long-term disability. It is a neurological deficit due to impaired blood supply to the brain areas caused by ischaemia or haemorrhage or occasionally both [1, 2]. Impaired blood supply to the brain results in a cascade of pathological processes that disrupt neurophysiological mechanisms and expression of Central Nervous System (CNS) biomarkers that eventually cause neuronal cell injury or death. When neuronal cells are injured, they discharge cytotoxic molecules that further injure or damage other apparently healthy neuronal cells [2–4]. This in turn creates a vicious cycle of cell injury and/or death that cause impairments in brain functions such as motor, sensory, and cognitive functions. Therefore, preventing or reducing the disruption of neurophysiological mechanisms and expression of CNS biomarkers by these pathological processes should be the target of treatment and rehabilitation following stroke. This may help prevent neuronal cell damage, improve neuronal cell homeostasis, and restore functions of the CNS.

One of the most promising rehabilitation techniques used for recovery of motor function after stroke is Constraint-Induced Movement Therapy (CIMT). The CIMT is a technique that comprises of massed task practice with the affected limb, constraint of the unaffected limb, and transfer package [5–7]. It has been reported to improve the use of limbs in daily activities and improve the quality, quantity, and precision of movement [8, 9]. However, these findings relate to the system or functional level of the nervous system. According to Cohen, to effectively understand the functions of the nervous system, it needs to be studied at the molecular and functional levels, and possibly also at other sublevels between them [10]. Consequently, current evidence has shown that repetitive functional activity or modulation of afferent inputs can induce growth, modification, degradation, and death of neuronal cells which can help the CNS to recover from injury [11, 12].

In practice, however, understanding the precise biomarkers of the process of recovery after stroke may be difficult due to differences in patients' presentations and the recovery processes [13]. The aim of this article is to review some of the CNS biomarkers CIMT targets after stroke and their correlations with motor function outcomes in both humans and animals (since animal studies serve as basic foundations for studies in humans). This will help us further understand the biomarkers of motor recovery following stroke, and possibly help researchers and clinicians identify the type of patients CIMT is more suitable for. A biomarker can be a gene, a naturally occurring molecule, or a particular characteristic by which a physiological or pathological process or disease can be identified [14]. It includes imaging biomarkers that are identified using computed tomography, positron emission tomography, transmagnetic stimulation, and magnetic resonance imaging; and molecular biomarkers such as a particular protein or gene expression [15–17]. The search engine PubMed was used using the search term Constraint-Induced Movement Therapy. The biomarkers extracted from the CIMT studies obtained from PubMed were further searched on Google Scholar in order to obtain more information. The characteristics of some of the reviewed studies in humans are presented in Table 1.

## 2. The Biomarker Effects of CIMT

### 2.1. Increased Perfusion (Cerebral Blood Flow) of Motor Areas

Cerebral blood flow (CBF) is important to help maintain an adequate supply of oxygen and glucose to the brain [18, 19]. Glucose and oxygen are essential for the growth, maturation, and functions of neurons [19]. An adequate supply of glucose is an important factor for cerebral metabolism which is also a major determinant of CBF [18, 20, 21]. CBF decreases shortly after stroke, and this disrupts the relationship between CBF and cerebral blood volume [18, 22, 23]. Additionally, decreased CBF results in hypoperfusion and inadequate supply of oxygen and glucose to the brain, following which neurons begin to get damaged or die [18]. CIMT can, however, help increase perfusion in the motor areas of the brain. According to Könönen et al., CIMT results in increased perfusion of motor areas in both the affected and nonaffected hemispheres [24]. In the affected hemisphere, the areas with increased perfusion were the precentral gyrus, superior frontal gyrus, premotor cortex, and frontal cortex; whereas in the unaffected hemisphere, the areas were the cingulate gyrus and frontal gyrus. The areas in which there was increased perfusion receive and integrate information from sensory systems and plan movement execution [24, 25].

Similarly, increased cortical activation bilaterally following CIMT may serve as an indication of increased cerebral perfusion [26]. In addition, increased perfusion and cortical activation bilaterally may also serve as signs of an increased cortical map. When the cortical map increased in size by recruitment of nonaffected brain areas, motor function was observed to be significantly improved [27, 28]. Interestingly, increased perfusion following the use of CIMT was associated with gain in motor function [29]. However, this was a case report on children with hemiplegic cerebral palsy, and therefore, there is still a need to determine this association in patients with stroke. Furthermore, CIMT should start very early after stroke to help improve CBF since persistence of ischaemia up to 24 hours will lead to lesion expansion and subsequent neuronal cell damage [30, 31]. In humans, there is little evidence on the safety of the use of CIMT very early after stroke [32].

### 2.2. Improved Brain Glucose Metabolism

The brain exclusively uses glucose for energy metabolism which is supplied under normal circumstance by CBF [18, 19]. Metabolism on the other hand determines the direction of blood flow [15, 16]. In the brain, local blood flow regulation is significantly related to intracellular partitioning of glucose transport and metabolism [19]. The glucose supplied by CBF is also required to provide the precursors for neurotransmitter synthesis and the ATP to stimulate their actions [33]. These indicate that the whole process is a sort of positive feed forward mechanism in which improved glucose metabolism affects local CBF, and increased CBF provides the brain cells with oxygen and glucose necessary for their growth and function.

When there is increased CBF in motor areas, movement planning and execution improves [24]. In addition, the cerebral metabolic rate of glucose (CMRglu) is significantly correlated with functional recovery [34]. However, brain glucose metabolism is impaired after stroke [35]; and as this affects local CBF, brain cells will be deprived of essential nutrients such as glucose and oxygen. The deprivation of these vital nutrients can also be confounded by lack of glucose and oxygen stores in the brain [19, 36]. Therefore, the reduced delivery of glucose and oxygen during brain ischemia will cause ATP depletion, which will in turn trigger processes leading to cell death [37].

To improve glucose metabolism following stroke, CIMT has been used. According to Li et al., after two weeks of CIMT in animals, the CIMT group showed improved glucose utilization indicated by lower standardized uptake values (SUVs) in the ipsilesional cingulate, motor, and somatosensory cortices and in the anterodorsal hippocampus compared to the control group [38]. It was also noted that in the AcbCore shell and cortex insular of the contralateral hemisphere responsible for rewarding behaviour and motor control, there were higher SUVs in the CIMT group than in the control. Increase in glucose utilization improves brain functions, and in a brain with normal oxygen concentration, aerobic glucose oxidation is solely responsible for over 95% of ATP production [35]. Improved glucose metabolism can also determine the direction of CBF [19, 20], and following the use of CIMT, improved perfusion was noted in motor areas responsible for movement control [24]. However, studies are still needed in humans to determine whether CIMT improves brain glucose metabolism. Additionally, since metabolic changes including decreased glucose metabolism occurs within the first 24 hours after stroke [39], it is important that CIMT is started as early as possible after stroke to prevent further neuronal cell damage. However, this should be done with caution, as hyperglycaemia during acute brain ischaemia in animals has been shown to exacerbate brain injury [40, 41].

### 2.3. Increased Brain-Derived Neurotrophic Factor (BDNF) Expression

Following stroke in animals, production of neuroblasts, which are undifferentiated precursors of neurons, significantly increases especially in the subventricular zone (a structure in the lateral walls of the lateral ventricles) [42]. Differentiation and survival of these neuroblasts are promoted by the Brain-Derived Neurotrophic Factor (BDNF) [43, 44]. The BDNF is a secretory growth factor that supports the survival of existing neurons and promotes synaptogenesis and differentiation of new neurons [45–47]. When new neurons survive and differentiate, synaptic integration occurs [48, 49]. Unfortunately, during the acute phase of stroke (first day after stroke), the concentration of BDNF decreases and this is associated with low functional neurological status [50]. However, BDNF can be secreted in response to an activity [46, 51, 52]. For instance, in humans, a combination of two weeks CIMT and repetitive transmagnetic stimulation (rTMS) increased the concentration of BDNF and its precursor, matrix metalloproteinase-9 (MMP-9) in participants who were more than one month poststroke [53]. Similarly, in animals, CIMT alone increased the expression of BDNF [54]. In the animal study, CIMT started on the first day after stroke; whereas in the human study, it started more than a month after stroke. These findings seem to suggest that CIMT alone may be sufficient to increase the concentration of BDNF when it is administered very early poststroke. In the chronic stage, CIMT may need to be combined with neurostimulation techniques such as rTMS to increase the concentration of BDNF.

In addition, BDNF genotype has been shown to interact with rehabilitation intervention and functional status in patients with stroke [55]. This seems to suggest that BDNF may be an important biomarker for CIMT and that a particular group of patients who possess this allele may benefit more from CIMT due to their genetic predisposition. Thus, researchers need to characterize their patients in planning rehabilitation intervention using CIMT in order to know who will benefit the most. Researchers also need to possibly combine CIMT with other rehabilitation strategies/techniques for those groups of patients who are less likely to benefit from the sole use of CIMT due to their genetic predisposition. However, more studies in humans are needed to verify the potential effect of CIMT on BDNF concentration in people with stroke.

### 2.4. Decreased Level of *γ*-Aminobutyric Acid (GABA)

*γ*-Aminobutyric acid (GABA) is the dominant neurotransmitter in the human brain that has an inhibitory effect and is involved in the process of neuroplasticity [56, 57]. Following stroke, the delicate balance between it and the excitatory neurotransmitters, such as glutamate and aspartate, becomes disrupted [58, 59]. Glutamate and aspartate levels become raised in the first six hours, while GABA and glycine levels are at their peak after the first day following ischaemic injury [59]. From then on, GABA level may remain high or at its peak, suggesting that treatment or rehabilitation to reduce the level of GABA should begin very early after stroke.

Decreased GABA level is a precursor to long-term potentiation, an important cellular biomarker for nervous system activity and motor learning [60, 61]. Therefore, decreasing GABA level should be an important goal of rehabilitation. Accordingly, Blicher et al. reported a decreased level of GABA in relation to creatine after two weeks of CIMT in subacute and chronic stroke patients [56]. They also showed that the decrease had good correlation with improvement in motor function. The correlation between motor function and a decreased level of GABA could also be a result of improved brain glucose metabolism; CIMT has been shown to improve brain glucose metabolism [38]. Effective brain glucose metabolism is required to provide the precursors for synthesis of neurotransmitters [19]. Therefore, it is possible that CIMT results in increased synthesis of excitatory neurotransmitters such as glutamate which may counteract the dominant inhibitory effect of GABA in the brain. Reducing GABA-mediated inhibition resulted in improved forelimb function in mice [62]. However, this claim needs to be further determined or investigated. Additionally, the effect of CIMT on GABA after stroke requires further studies in humans.

### 2.5. Increased Expression of Growth-Associated Protein 43 (GAP-43)

Growth-Associated Protein 43 (GAP-43) is involved in the regulation of axonal outgrowth, synaptic plasticity, and learning and memory functions [63]. After an injury to the CNS such as stroke, there is upregulation of GAP-43 which occurs between seven days and three weeks after the ischaemic injury [64, 65]. The upregulation is positively related to long-term potentiation (LTP), which is a precursor for neural plasticity [66]. In an animal model, CIMT that was initiated on the first day poststroke resulted in increased expression of GAP-43 [54]. In contrast, CIMT performed seven days after stroke produced different results [54, 67]. In the study by Ishida et al., CIMT did not yield increased expression of GAP-43 [54]; but in the study by Zhao et al., it yielded increased expression of GAP-43 [67].

Possibly, the difference in the results of the two studies could be because of the types of stroke. Whereas in the study by Ishida et al. the stroke was due to subcortical haemorrhage induced by injection of collagenase, in the study by Zhao et al., the stroke was due to focal cerebral ischaemia induced by intracerebral injection of endothelin-1. Ischaemic stroke usually has a better outcome than the haemorrhagic type [68]. Therefore, since GAP-43 is normally upregulated seven days following ischaemic injury [65], early CIMT (starting on the first day after the ischaemic injury) may help raise GAP-43 level early on to help in the process of neural plasticity through induction of long-term potentiation. This is important since at approximately six days after ischaemic injury, lymphocytes and macrophages accumulate in the perivascular vicinity [69]. Accumulation of these cells may further damage neuronal cells. However, there seems to be no study as yet in humans reporting on the role GAP-43 has on the recovery of motor function in stroke following CIMT.

### 2.6. Increased Expression of Hypoxia-Inducible Factor-1*α* (HIF-1*α*) and Vascular Endothelial Growth Factor (VEGF)

In rats, CIMT performed for two weeks resulted in a significant increased total length of microvessels and number of bromodeoxyuridine+ (BrdU+)/NeuN+ double-labeled cells [70]. These changes correlated well with increases in Hypoxia-Inducible Factor-1*α* (HIF-1*α*) and Vascular Endothelial Growth Factor (VEGF) expressions and a decrease in Factor Inhibiting HIF-1*α* (FIH-1*α*) expression. Hypoxia-Inducible Factor-1*α* (HIF-1*α*) is a key regulator in hypoxia, and due to the functions of its downstream genes, it has been suggested to be an important player in neurological outcomes following ischemic stroke [71]. The downstream genes include those that promote glucose metabolism, angiogenesis, erythropoiesis, and cell survival [72, 73]. Additionally, HIF-1*α* is a key regulator of VEGF [74]. Furthermore, HIF-1*α*, VGEF, CBF, and glucose metabolism are part of the system that restores the oxygen supply to tissues when blood circulation is inadequate such as in hypoxic conditions like stroke [24, 38, 75]. This synergy may perhaps be the one that helps prevent cell death in stroke and improve CNS functions following the use of CIMT. However, it is not yet known whether these findings can be related to humans, as the effect of CIMT on HIF-1*α* and VGEF has not yet been determined in the human species.

### 2.7. Downregulation of Phosphorylated Extracellular Regulated Protein Kinase Expression

The Extracellular Regulated Protein Kinase (ERK) pathway, which is a subfamily of mitogen-activated protein kinases, is a key player in cerebral ischaemia [76, 77]. It plays roles in the mediation of a number of physiological and pathological processes in cells, such as proliferation and differentiation [77, 78]. Additionally, p-ERK plays roles in the activation of inflammation response and oxidative stress during early poststroke [77]. During this period, specifically up to 24 hours, infarct volume increases as the ischaemia persists [29, 30]. Secondary changes such as early phase vasogenic oedema and a later phase with inflammatory infiltration develop after 24 hours [79–81]. This is the period when p-ERK plays its role.

Inhibition of ERK phosphorylation (p-ERK) results in reduced infarct volume and protection of neural cells [82]. This is achieved when its expression is downregulated. Consequently, use of CIMT in rats significantly lowered the expression of p-ERK in the bilateral cortex and hippocampi similar to the sham group levels than that in the model group [82, 83]. The authors argued that these data suggest that functional recovery after CIMT may be related to decreased expression of phosphorylated extracellular regulated protein kinase in the bilateral cortex and hippocampi. Furthermore, since the pathophysiological changes such as the lesion expansion and the inflammatory responses to ischaemia occur with the first few days after stroke, CIMT needs to start very early poststroke to help downregulate p-ERK. However, these findings need further investigation in human stroke.

### 2.8. Upregulation of Stromal Cell-Derived Factor 1 (SDF-1)

Stromal Cell-Derived Factor 1 (SDF-1) is upregulated following stroke and is especially localized to the ischaemic penumbra [84]. Following the use of CIMT in rats, motor function significantly improved with a corresponding increase in SDF-1 protein levels in the cortex and dentate gyrus [85]. An increased level of SDF-1 is positively correlated with an increased concentration of endothelial progenitor cells (EPC), which are important for vascularization of brain tissues [86]. Vascularization helps provide the brain tissue with oxygen and glucose which are important for the healthy growth and function of brain cells [18, 19]. Additionally, upregulation of SDF-1 is inversely correlated with the volume of the lesion (as SDF-1 level increases, the volume of the lesion decreases) [86]. This is because SDF-1 is noted to be localized around the ischaemic penumbra, and its role is to prevent the lesion from expanding by improving vascularization of the area. Since the infarct volume expands up to 24 hours when ischaemia persists [30, 31], CIMT needs to be started very early after stroke in order to help increase the level of SDF-1 and possibly enhance vascularization and the attendant neurogenesis. Accordingly, in the study by Zhao et al., CIMT was initiated early on poststroke (at 7 days after stroke) [85]. However, as yet, there seems to be no study that reported on the effect of CIMT on SDF-1 in humans.

### 2.9. Increased Numbers of *Δ*FosB-Positive Cells

The *Δ*FosB-positive cells are transcription factors responsible for mediating long-term neural and behavioural plasticity [87]. They encode proteins necessary for cell proliferation, differentiation, and transformation [88]. Therefore, they serve as biomarkers for use-dependent neuronal activity [89] and can be activated by repetitive stimulation [90]. Previously, enriched environment training was shown to increase expression of *Δ*FosB-positive cells in the perilesional cortex [91].

Similarly, in rats, early CIMT (initiated on the first day after stroke) was associated with greater numbers of *Δ*FosB-positive cells in the ipsilesional sensorimotor cortex layers II-III and V [54]. Therefore, starting CIMT within the first 24 hours after stroke may help raise the number of *Δ*FosB-positive cells and prevent further pathological processes that will lead to cell death. However, this effect has not yet been reported in humans, and therefore, future CIMT studies should look at the possible role of CIMT in modulating *Δ*FosB-positive cells. Additionally, the effect seen even in animals was only in haemorrhagic stroke. Thus, even though the pathophysiology of haemorrhagic stroke may eventually take the form of ischaemic stroke, studies in the latter are equally needed. This is especially important because the latter type of stroke is more common and tends to have better prognosis than the former [1, 68].

### 2.10. Improved Cortical Activation

Cortical activation refers to the stimulation of the brain structures responsible for its function. Following CIMT, a significant increase in primary motor cortex (PMC) and somatosensory cortex (SMC) activation in both ipsilesional and contralesional hemispheres has been reported [92–97]. In each one of the following studies, increased activation occurred during the acute stage [93] and subacute stage [94]. In four studies, increased activation occurred during the chronic stage [92, 95–97]. These findings seem to suggest that increasing cortical activation following CIMT is possible at any stage of stroke. However, during the chronic stage, CIMT was combined with either tDCs or bilateral arm training [97, 98], probably suggesting that during this period, a combination of CIMT with another rehabilitation therapy may be required to increase cortical activation.

Similarly, it was also suggested that in chronic stroke patients, CIMT should be combined with electrical stimulation since a close relationship was found between increased hand function with peak changes in activation within the ipsilesional SMC following the combination of these two therapy techniques [95]. In contrast, in acute stroke patients, CIMT alone may be used. According to El-Helow et al., there were improved resting motor threshold (RMT), central motor conduction time (CMCT), and amplitude of motor-evoked potentials (MEPs) which are evidences of increased cortical activation when CIMT alone was used in acute stroke patients [99]. However, evaluating cortical activation depends on the type of probe used. In four studies, transmagnetic stimulation (TMS) was used [92, 93, 98, 99]. The TMS measures motor-evoked potentials (MEPs), neuroelectrical signals produced by the spinal cord and peripheral muscles when the brain is stimulated. In three studies, functional magnetic resonance imaging (fMRI) was used [94–96]. The fMRI records blood oxygen-level-dependent (BOLD) signal activity changes [100]. According to Liew et al., when evaluating cortical activation, it is better to combine the use of more than one probe technique [13]. Interestingly, one of the cited studies combined the use of TMS and fMRI and reported a significant positive correlation between the two probe techniques [96].

In addition, the severity of the patient's motor impairment before intervention is a factor to note in determining an increase in cortical activation. According to Könönen et al., increased cortical activation is higher in those patients with poor motor ability before intervention with CIMT or in those whose motor ability improved remarkably after the intervention [96]. Similarly, when the pyramidal tract (PT) is intact, improvement in motor function initially correlates with a decrease in activation in SMC and then an increase after 6 months [101]. When PT is affected, improvement consistently correlates with an increase in a lateral extension of SMC. Improvement in cortical activation has been shown to correlate well with motor functions [26, 27, 93, 96]. These may be because those with mild and/or moderate impairment in motor function have activation patterns similar to apparently healthy individuals. However, different regions of the brain may be activated differently following CIMT as different patients show different patterns of activation [26, 97, 101]. Patients with chronic stroke usually show a bilateral pattern of activation [13]; this is probably because of the recruitment of areas distant to or interconnected with the areas of the lesion. Those with greater motor impairment display increased fMRI activity in the contralesional hemisphere [102, 103], but those with better motor function show normal activation patterns in the ipsilesional hemisphere [104, 105].

Another variable which needs to be considered when attempting cortical activation is the use of constraint. This is because it has been reported that restriction of the contralesional limb led to decreased cortical activation in people with right hemispheric stroke especially in the cortical and basal ganglia motor areas [106]. In those with left hemispheric stroke, a decreased activation pattern was noted even in the absence of limb restriction, which can induce an additional cortical silencing (which is an indicator of cortical disinhibition). Since use or no use of constraint did not show any difference in improvement in motor function following CIMT [107], these findings seem to suggest that the use of a constraint may not be necessary, though it has been argued that it may help to increase the number of task repetitions which is essential for motor recovery [108]. One of the clinical significance of these findings is that they further affirm that what matters the most is the frequency of repetition of the functional tasks during CIMT.

### 2.11. Downregulation and Upregulation of Intracortical Inhibition

Output from the cortex is dependent upon the balance between different inhibitory and facilitatory circuits in the brain [109, 110]. Stroke results in intracortical inhibition in the affected hemisphere [111]. This intracortical inhibition was shown to be significantly downregulated or upregulated in the ipsilesional hemisphere compared to the contralesional hemisphere after a few weeks of CIMT [111, 112]. The amount of disinhibition was related to the degree of spasticity [112]. Additionally, evaluation of inhibition depends on the type of probe used. In the study by Liepert et al., TMS and fMRI were used to evaluate intracortical inhibition [111], while in the study by Liepert, TMS was used to evaluate intracortical inhibition [112]. Thus, combining TMS and fMRI in evaluating cortical activity should be encouraged in studies since they measure motor-evoked potentials (MEPs) and record blood oxygen-level-dependent (BOLD) signal activity changes, respectively. These will provide better outcomes for studying brain activity. However, further studies are still needed to determine which of the upregulation or downregulation of intracortical inhibition is significantly related to improvement in motor function.

### 2.12. Modulation of Interhemispheric Imbalance

Under normal circumstances, interhemispheric inhibition plays an important role during performance of a unimanual task such as brushing one's teeth or writing. Following stroke, cortical excitability of the ipsilesional hemisphere tends to be lower than that of the contralesional hemisphere [98, 113]. Thus, the contralesional hemisphere may tend to inhibit the ipsilesional hemisphere. However, rehabilitation therapy can help either counteract or reduce this inhibition. A combination of tDCs with CIMT reduced interhemispheric imbalance between the affected and unaffected hemispheres when motor evoked potential values were compared between baseline and after intervention [98, 114, 115]. In one study, what exactly happened was that the ability of the ipsilesional hemisphere to counter inhibition by the contralesional hemisphere improved even though the inhibition by the contralesional hemisphere did not change significantly [115].

In addition, on modulation of interhemispheric imbalance, two factors need consideration: the stage of stroke and the type of probe used. In one study, the participants were within acute stage of stroke and the probe used was fMRI-guided TMS [114]. In contrast, in three studies, the participants were within chronic stage of stroke and the probe used was only TMS [98, 114, 115]. In all these studies, CIMT was combined with brain stimulation, which seems to suggest that a combination of CIMT with brain stimulation is required to reduce interhemispheric inhibition following stroke. However, since CIMT was administered in these studies for a period not longer than five weeks, it is possible that when CIMT alone is administered for a longer period, interhemispheric inhibition could also be modulated. This is needed since even in healthy individuals, a combination of tDCs with motor training showed a decrease in cortical excitability in the dominant hemisphere and a decrease in transcallosal inhibition from the dominant to nondominant hemisphere [116]. Additionally, studies that combine different probe methods are needed to help elucidate neural activities in both hemispheres.

### 2.13. Increased Cortical Map Size

Motor map size is the extent to which control of movement of a part of the body is represented in the brain. It is in essence a measure of the size of the area that represents or controls movement of a part of the body. Motor map size shrinks after stroke, but it can be enlarged with the use of rehabilitation therapy. Accordingly, following the use of CIMT for 10 days, motor map size increased significantly in the experimental group compared with that of the control and the increase correlated well with motor function [27, 28]. Specifically, the correlation was higher and more significant between baseline and four months postintervention [28]. Additionally, the cortical silent period which is a measure of disinhibition increased significantly in the experimental group, though it was not significantly correlated with motor function. This seems to suggest that an increase in motor map size is more important in motor recovery than decreased disinhibition in the ipsilesional cortex. This is probably because the extra map could carry the brunt of control of the areas represented by the cortex.

On increase of motor map size, three issues are worthy of note. One, a study reported that there was no significant difference in motor map size and motor function between early and delayed CIMT at four months follow-up [28]. This is because, at the time of the follow-up, the participants in both the experimental and the control groups would have been in chronic stage. Secondly, no significant difference in the silent period was found between the CIMT group and the control group recently [117]. Thirdly, a posterior shift in the center of gravity (COG) was observed in the late group from baseline to four months, and that the shift was more in the late group compared to the early group; in the same way, a considerable shift in mean COG was reported earlier in chronic stroke patients [92]. Thus, it seems that in stroke patients, it is highly likely that CIMT improves the recovery of motor function by recruiting adjacent brain structures than by improving cortical activation especially in chronic stroke patients. According to Sawaki et al., the late CIMT group showed decreased excitability in the ipsilesional hemisphere [118]. Similarly, in monkeys, delayed training resulted in a decrease in spared hand representation [119], probably affirming my previous claim. However, these studies reporting on increased cortical map size used only TMS which is a measure of evoked potentials. Therefore, future studies should use a combination of different probe techniques to enable the provision of more cogent evidence on increased cortical map size.

### 2.14. Changes in the Effective Connectivity of Primary Motor Cortex

The primary motor cortex is a part of the motor cortex involved in the control and execution of voluntary movement [25]. Under normal circumstance, it is effectively connected to other parts of the motor cortex such as the premotor, posterior parietal, and primary somatosensory cortices and to other parts of the brain such as the cerebellum and thalamus that play roles during movement control and execution. However, the effective connectivity of the primary motor cortex may get altered after stroke [120].

When the effective connectivity of the primary motor cortex is altered, it can be effectively reconnected with rehabilitation therapy. In a group of patients with chronic stroke, two weeks of CIMT showed changes in the local response of TMS in the ipsilesional and contralesional primary motor cortex (M1); changes in the strength of interhemispheric connectivity between M1s; and changes in the effective connectivity of the ipsilesional and contralesional M1s with the nonprimary motor areas, the basal ganglia, and the motor nuclei of the thalamus [121]. Thus, improving effective connectivity of the primary motor cortex of the ipsilesional and contralesional hemispheres, between the ipsilesional primary motor cortex, and different areas of the brain is an important mechanism through which CIMT aids in the recovery of motor function after stroke. This is because motor function is controlled by the motor system that comprises of a series of cortical and subcortical areas interacting via anatomical connections [122].

### 2.15. Structural Changes

Anatomical changes are important biomarkers for recovery after stroke. In animals, abundant dendritic arborization of pyramidal neurons in the sensorimotor area was noted following the use of CIMT [50]. Abundant dendritic arborization of pyramidal neurons helps in the formation of new synapses which results in improved neuronal communication and function. Similarly, three hours of CIMT for 10 consecutive days resulted in increased gray matter in sensory, motor, and hippocampi areas in both ipsilesional and contralesional hemispheres in humans [123]. Gray matter consists of neuronal cell bodies and synapses which are important for functions of the CNS. However, it is important to note that the process of cerebral reorganization is a slow one and may take months to complete [124].

## 3. Conclusion

CIMT facilitates and enhances neuronal cell homeostasis. In animals, this is achieved through improved perfusion of motor areas and brain glucose metabolism; increased expression of BDNF, SDF-1, HIF-1*α*, VEGF, and GAP-43; increased number of *Δ*FosB-positive cells; and decreased levels of GABA and p-ERK among others. In humans, there are so far a few studies reporting on improved perfusion, decreased level of GABA, and a potential interaction between increased level of BDNF and motor function; but a number of studies reported on increased cortical activation and motor map size, increased and decreased intracortical inhibition, and decreased transcallosal or interhemispheric inhibition of the ipsilesional hemisphere. Additionally, most of the findings on the biomarkers such as increased cortical activation and map size, SDF-1 expression, and greater numbers of *Δ*FosB-positive cells, indicate that it is important that CIMT is initiated early poststroke. However, implementation of CIMT has been very difficult during early poststroke in humans and as such there are still just a few studies related to this area. Furthermore, there is limited evidence that CIMT is more effective when it is combined with other therapies such as tDCs in chronic cases of stroke. It should also be noted that these effects are mostly in ischaemic stroke. Thus, studies in humans are needed to further elucidate the biomarkers for recovery of motor function following CIMT.

### 3.1. Implications for Research and Practice

The fact that there is good positive correlation between increased cortical map size and motor function improvement and between increased cortical activation and motor function improvement shows that CIMT has a strong theoretical basis. However, further research is needed to characterize those who have genetic affinity for improvement considering the BDNF gene and possibly other genetic constituents. Secondly, more rigorous studies with adequate sample sizes are needed to verify the discussed evidence especially on the molecular biomarkers in humans. Clinicians also need to create algorithms to be able know how much CIMT is required to produce effects on these biomarkers based on the patient's presentations and the likely duration of treatment and prognosis.

## Figures and Tables

**Table 1:** Characteristics of some of the reviewed studies.

Study	Design	Stage of stroke	*N*	Duration of therapy	Constraint	Additional therapy	Outcome measures
Wang et al. (2012)	Self-control experiment	Subacute	5	CIMT for 4 hours, 20 minutes, 5 times a week for 4 weeks	Nil	4 hours of lower limb loading exercise (Bobath) divided into 2 sessions per day for 6 weeks	MWS, BBS, fMRI
Blicher et al. (2014)	Clinical controlled trial	Subacute and chronic	41	CIMT for 2 weeks in the experimental group	Mitt for 90% of waking hours	Nil	WMFT, MRI, mRS
Könönen et al. (2005)	Pretest Posttest	Chronic	12	CIMT for 6 hours per day for 10 days	Lightweight sling worn during the exercise and for 10 hours per day	Nil	WMFT, MRI, SPECT
Di Lazzaro et al. (2014)	RCT	Acute	14	40 mins and 30 s tDCs for experimental and control groups, respectively, for 5 days	Resting sling for 90% of waking hours	Standard physical therapy protocol	9HPT, MAL, NIHSS (motor), hand grip, mRS
Rijntjes et al. (2011)		Chronic	12	6 hours of CIMT/day for 2 weeks	Splint for 90% of the waking hours	Nil	Same as above plus TMS
Boake et al. (2007)	RCT	Acute	23	3 hours of CIMT and traditional therapy for CIMT and control groups, respectively	Constraint for 90% of the waking hours		FM, TMS, GPT, MAL
Chouinard et al. (2006)	Pretest Posttest	Chronic		4 hours of CIMT for 2 weeks	Wearing of mitt at home	Nil	TMS/PET
Ro et al.(2006)	RCT	Acute	8	3 hours of CIMT and traditional therapy, respectively, 6 days a weeks for 2 weeks	Constraint for 90% of the waking hours	Nil	TMS, MAL

Key: MWS=Mean walking speed; BBS=Berg balance scale; fMRI=Functional magnetic resonance imaging; SPECT=Single-photon emission computerized tomography; fMRI=functional magnetic resonance imaging; FM=Fugl Meyer; ARAT=action research arm test; WMFT=Wolf motor function test; MAL=motor activity log; MRI=magnetic resonance imaging; mRS=modified Rankin scale; 9HPT=nine-peg hole test; NIHSS=National Institute of Health Stroke Scale; TMS=transmagnetic stimulation; PET=positron emission tomography; GPT=groove peg test.
